# Comparison of Intraperitoneal Irrigation With Sodium Bicarbonate Versus Normal Saline in Reducing Pain After Operative Laparoscopy: A Randomized Controlled Trial

**DOI:** 10.7759/cureus.47686

**Published:** 2023-10-25

**Authors:** Ankita Agarwal, Rinchen Zangmo, Deepali Garg, Kallol K Roy, Avir Sarkar, Anshul Kulshreshtha, Ashmita Saha

**Affiliations:** 1 Obstetrics and Gynecology, All India Institute of Medical Sciences, New Delhi, New Delhi, IND

**Keywords:** salpingectomy, cystectomy, adhesiolysis, post-operative pain, laparoscopy

## Abstract

Background

It has been postulated that sodium bicarbonate can reduce postoperative pain by neutralizing the acidic peritoneal environment created by carbon dioxide. It also prevents phrenic nerve damage and peritoneal irritation. The present study is a randomized controlled trial aimed at studying the effects of sodium bicarbonate in reducing postoperative pain in laparoscopic gynecological surgeries.

Materials and methods

This was a single-center, prospective, two-arm, double-blinded randomized control trial in which intraperitoneal irrigation with sodium bicarbonate was compared with normal saline in operative laparoscopy. Group I (intervention group) consisted of 40 patients who received intraperitoneal sodium bicarbonate, and Group II (control group) consisted of 40 patients who received normal saline. All procedures were conducted under general anesthesia. Postoperative pain scores were compared between intervention and control groups.

Results

The most common indication of laparoscopy was infertility. There was no difference in the duration of surgery between the two arms (p=0.27). The mean value of the visual analog scale (VAS) score at the shoulder tip was found to be significantly reduced in the intervention group at two hours (p=0.02), four hours (p=0.0009), and 12 hours (p=0.0002) after surgery. The mean VAS score at the abdomen and port sites was also found to be significantly reduced in the intervention group in the first 24 hours after surgery (p<0.05). With the increase in the time period from surgery, the mean VAS scores decreased in both intervention and control groups.

Conclusion

Intraperitoneal irrigation with sodium bicarbonate is beneficial in reducing postoperative pain in operative laparoscopy. However, multicenter randomized trials with a greater number of participants will be helpful to confirm the findings.

## Introduction

Laparoscopy is a minimally invasive surgery that has rapidly evolved to be a major surgical modality over the past 30 years, representing one of the most significant advancements in the surgical field [1]. Initially, it was used only for diagnostic purposes. In 1910, the first laparoscopy was performed by Hans Christian Jacobaeus of Sweden, using a Nitze cystoscope, a hollow tube with a candle, to illuminate the peritoneal cavity. Advanced operative laparoscopic procedures were introduced in Germany in the 1970s. Today, laparoscopy can execute nearly all surgical procedures that are typically done via open surgery [1,2]. It is useful in evaluating patients with chronic pelvic pain, infertility, recurrent pregnancy loss, menstrual irregularity, and adnexal mass. Laparoscopy helps identify conditions such as endometriosis, pelvic adhesions, uterine fibroids, hernias, and adnexal masses. It can also be used to treat ovarian cysts, endometriosis, fibroids, ectopic pregnancies, sterilization issues, urinary incontinence, and pelvic support problems like uterine prolapse, which previously required large incisions [2]. Laparoscopic procedures offer several advantages: reduced blood loss, decreased postoperative pain, early recovery, lower morbidity, shorter hospital stays, fewer chances of developing adhesions, reduced risk of bowel obstruction, and smaller incisional scars, leading to superior cosmetic results [3]. However, postoperative pain following laparoscopy can be significant, causing discomfort to patients. This pain can delay a patient's recovery, cause delayed mobilization, increase analgesic requirements, prolong hospital stays, raise hospital costs, elevate postoperative morbidity, and overall delay recovery. Pain can occur at the back, shoulders, and both upper and lower abdomen [4]. It is hypothesized that carbon dioxide (CO2), which is introduced into the peritoneal cavity to create pneumoperitoneum, creates an acid milieu by the dissolution of CO2 gas, which causes phrenic nerve damage and peritoneal irritation [4]. Phrenic nerve excitation can lead to prolonged shoulder tip pain [5]. Other suggested etiologies for pain include small blood vessel tears due to trauma, CO2 insufflation causing stretching of abdominal tissue, release of inflammatory mediators, or nerve traction [6]. Pain control techniques, including non-steroidal anti-inflammatory drugs (NSAIDs) and opioid analgesics, have been shown to be effective in the postoperative period. However, opioids can cause various adverse effects, such as constipation, nausea, vomiting, pruritis, respiratory depression, drowsiness, and urinary retention [7]. These opioid-related side effects may delay mobilization, increase the duration of hospital stay, and delay recovery, thereby diminishing the benefits of laparoscopic procedures. Therefore, there is a need to lessen the use of opioid analgesics. Excess use of analgesics is also not devoid of side effects. Other methods that have been studied include putting a gas drain under the diaphragm, active aspiration of gas, local anesthetic application on the skin and muscle of the wound, and intraabdominal irrigation with saline, bupivacaine, or bicarbonate [8]. As CO2 might have a key role in causing postoperative pain, washing out or neutralizing CO2 may play a role in reducing postoperative pain. It has been postulated that by neutralizing the acidic peritoneal environment that CO2 creates, bicarbonate can decrease postoperative pain. It can also protect the phrenic nerve from damage, and it reduces peritoneal irritation. The present study was a randomized controlled trial aimed to study the effect of sodium bicarbonate in reducing postoperative pain in operative laparoscopic surgeries.

## Materials and methods

It was a double-blinded, randomized controlled trial consisting of 80 participants (40 in each arm). Ethical clearance was obtained from the Institute Ethics Committee for Post Graduate Research, All India Institute of Medical Sciences, New Delhi (AIIMS, New Delhi) (IECPG-553/21.10.2020, AA-1/25.11.2020, RT-21/25.11.2020). All patients undergoing operative laparoscopy were recruited from the Gynecology outpatient department (OPD) at the AIIMS, New Delhi. The study was also registered in the Clinical Trials Registry - India: CTRI/2020/07/034995. All patients were informed about the procedure and informed written consent was obtained at recruitment. The study was conducted between July 2020 to June 2021 during the COVID-19 pandemic. The primary outcome measure was the comparison of effects on postoperative pain in intraperitoneal sodium bicarbonate versus normal saline groups.

Sample size calculation

According to an earlier study by Saadati K et al. in 2016, the mean +/- SD visual analog scale (VAS) score at 24 hours for shoulder tip pain between the sodium bicarbonate and saline group was 1+/-1.7 and 1.7+/-2, respectively. Assuming a similar observation to be made in the present study, the adequate sample size with an 80% power of study and a 5% level of significance came out to be 110 in each arm [7]. In view of the COVID-19 pandemic situation, it was unlikely to obtain the decisive number of patients. Therefore, in anticipation of a smaller number of patients visiting the OPD and after discussing with our departmental statistician, who is also a co-guide for the thesis, we decided to recruit a minimum of 30% of the sample size, i.e., 40 in each arm. Consequently, a total of 80 patients were recruited for this study.

Inclusion and exclusion criteria

We included women who required operative laparoscopic procedures (such as ovarian cystectomy, oophorectomy, ovarian drilling, salpingectomy, and adhesiolysis) during the study timeframe and were willing to provide written informed consent for the trial. We excluded women with a history of chronic use of opioids, NSAIDs, alcohol, and steroids. Additionally, patients with chronic kidney disease, diarrhea, renal tubular acidosis, chronic obstructive pulmonary disorders (COPD), or any known allergy to bicarbonate products were not considered.

Randomization

The randomization of participants was done using the statistical software Epi-Info version 7.0. Random generation of numbers (between one and eighty) was performed using the software. Two sets of envelopes were prepared. Each envelope displayed the generated number on its exterior, and inside each envelope was a label marked either "I" or "II." Patients who met the inclusion criteria were randomized to either group "I" or "II" based on these envelopes. When patients were scheduled for surgery, their corresponding envelope was opened, and the blinded solution "I" or "II" was administered based on the label inside the envelope. The necessary amount of solution was prepared and placed in a one-liter identical bottle for each patient. Each solution was coded as "I" or "II," a detail unknown to the investigator. Both the investigator and the patient were unaware of the specific solution type, ensuring that it was a double-blinded study.

Intervention and control groups

The intervention group (Group I, consisting of 40 participants) received intraperitoneal irrigation with sodium bicarbonate (50 ml of 7.5% sodium bicarbonate diluted in one liter of normal saline after the procedure). Group II was the control group (consisting of 40 participants) who received intraperitoneal irrigation with one liter of normal saline after the procedure. 

Data collection

A complete evaluation was done for each patient, taking into account their demographic profile, BMI, and eligibility criteria. A complete general physical and gynecological examination was done. All patients were interviewed and informed written consent was taken. All patients underwent preoperative investigations for fitness for administration of general anesthesia. In all patients, arterial blood gas (ABG) analysis was done both before and after the procedure to look for the acid base and pH changes. A nasopharyngeal sample for COVID-19 was obtained from all participants, and they proceeded with the procedure only after receiving a negative report. Appropriate COVID-19 protocol was followed to minimize the spread of COVID-19 infection.

Postoperative VAS scoring was conducted at 2, 4, 12, and 24 hours, as well as 1 week after the procedure to compare the effects on postoperative pain between the two groups. All surgeries were performed under general anesthesia, following a standard protocol. Anesthesia induction was achieved using propofol and fentanyl. Muscle relaxation was initiated with atracurium and maintained with either isoflurane, atracurium, or fentanyl, depending on the patient's individual profile and the anesthetist's choice. All laparoscopies were performed by a single operator. A standard operative method was used with a 3-trocar technique in all patients. Pneumoperitoneum was achieved with CO2 insufflation through a periumbilical trocar and maintained at 12 mmHg during the entire surgical procedure. The size of the telescope used was 10 mm. Two five-millimeter ancillary ports were placed under guidance. After performing the procedure, hemostasis was achieved. Intraperitoneal irrigation with 50 ml of 7.5% sodium bicarbonate diluted in one liter of normal saline (at 37°C) was done in the cases, and intraperitoneal irrigation with one liter of normal saline (at 37°C) was done in controls. In both groups, the fluid was completely aspirated before pneumoperitoneum deflation. After the procedure, the pneumoperitoneum was properly released. Local infiltration at port sites with 10 ml of 0.25% bupivacaine was done in both cases and controls before skin closure.

Operating time was noted in all cases in both groups. For postoperative analgesia, an injection of paracetamol 1 gm IV six hourly was given for the first 24 hours in both groups. Rescue analgesia in the form of injection of diclofenac sodium 75 mg intramuscularly (or injection of tramadol or paracetamol IV where diclofenac was contraindicated) was administered in patients needing additional analgesia. Patients who needed additional analgesic was recorded. The women were asked to rate the severity of their pain using a VAS score, with zero indicating no pain and 10 signifying unbearable pain. This was recorded at 2, 4, 12, 24 hours, and one week post-procedure. The pain was assessed at three distinct locations: port sites (three port site incisions), shoulder tips (both left and right), and the abdomen (excluding port sites and divided into each of the four quadrants). An independent evaluator, blinded to the specific intervention given to the patient, assessed the VAS score. Patients remained hospitalized for 24 hours post-procedure, allowing for VAS scoring up to that period to be conducted in the hospital. The VAS score at one week was recorded during the patient's visit for suture removal. The duration of the hospital stay was calculated from the end of the surgery until the patient's discharge.

VAS was used to assess postoperative pain in our study. VAS is a validated, subjective measure for acute and chronic pain. Scores are recorded by making a handwritten mark on a 10-cm line representing a continuum between "no pain" and "worst pain." Statistical analysis data was continuous and tested for normality assumption using appropriate statistical tests. Descriptive statistics, such as SD, and range values, were computed for normally distributed data, and the mean value was compared using the "independent t-test." For non-normally distributed data, median values and interquartile ranges were computed and compared using the non-parametric "Mann-Whitney test." A two-tailed probability of p < 0.05 was considered statistically significant. Given a level of significance of 5%, a total of 80 patients were recruited, with 40 in each group.

## Results

The two groups were comparable with respect to age, parity, indications for surgery, menstrual cycle, obstetric history, past medical history, and operating time. The p-value was not statistically significant (P>0.05) as depicted in Table 1.

**Table 1:** Comparison of patient details between both the groups.

Variables	Group I (n=40)	Group II (n=40)	P-value
Age in years	28.5	30.4	0.18
Parity			
0	37	33	0.62
1	2	5	
2	1	1	
3	0	1	
Indications for surgery			
Infertility for laparoscopy	21[52.5%]	22[55%]	0.85
Benign ovarian cyst	11[27.5%]	13[32.5%]	
Polycystic ovarian syndrome	5[12.5%]	2[5%]	
Chronic ectopic pregnancy	1[2.5%]	1[2.5%]	
Hydrosalpinx	2[5%]	2[5%]	
Menstrual Cycle			
Regular	32 [80%]	35 [87.5%]	0.36
Irregular	8 [20%]	5 [12.5%]	
Obstetric history			
Nulliparous	37[92.5%]	33[82.5%]	0.39
Vaginal delivery	1[2.5%]	2[5%]	
Previous one caesarean	2[5%]	5[12.5%]	
Past history			
Non-significant	35[87.5%]	33[82.5%]	0.49
Previous surgery	3[7.5%]	6[15%]	
Tuberculosis	2[5%]	1[2.5%]	
Operating time			
Mean	30.5 [± 3.9]	31.7[±5.6]	0.27
Minimum	24	23	
Maximum	38	42	

Mean values of the VAS score for shoulder tip pain at 2, 4, and 12 hours were calculated and compared between the two groups and were found to be statistically significant (P<0.05). Similarly, after completion of the surgery, the pain score was calculated for the abdomen, excluding port sites, using VAS at 2, 4, 12, and 24 hours, which were calculated and compared between the two groups and found to be statistically significant (P<0.05). Pain score was also calculated for the port sites at 2, 12, and 24 hours, compared between the two groups, and found to be statistically significant (P<0.05), as shown in Table 2. 

**Table 2:** VAS scores at 2, 4, 12, 24 hours, and 1 week for both groups who underwent adhesiolysis. VAS: Visual analog scale.

Group		VAS 2h	VAS 4h	VAS 12h	VAS 24h	VAS 1 week
Shoulder tip						
I	N	40	40	40	40	40
	Mean [±SD]	5.8 [±0.8]	4.8 [±0.7]	3.2 [±1.1]	2.0 [±0.7]	1.4 [±0.4]
II	N	40	40	40	40	40
	Mean [±SD]	6.3 [±1.1]	5.2 [±0.9]	4.1 [±0.9]	2.4 [±0.9]	1.4 [±0.5]
Results	P-value	0.02	0.0009	0.0002	0.06	0.65
Abdomen excluding port sites						
I	N	40	40	40	40	40
	Mean [±SD]	6.9 [±0.5]	5.2 [±0.6]	3.4 [±0.6]	2.0 [±0.7]	1.3 [±0.4]
II	N	40	40	40	40	40
	Mean [±SD]	6.9 [±0.6]	5.6 [±0.7]	4.1 [±0.8]	2.5 [±0.9]	1.4 [±0.5]
Results	P-value	0.85	0.003	<0.001	0.01	0.17
At port sites						
I	N	40	40	40	40	40
	Mean [±SD]	6.1 [±0.6]	5.4 [±0.5]	3.2 [±1.1]	1.8 [±0.7]	1.2 [±0.4]
II	N	40	40	40	40	40
	Mean [±SD]	6.5 [±0.9]	5.8 [±1.0]	4.2 [±0.9]	2.4 [±0.7]	1.4 [±0.5]
Results	P-value	0.01	0.053	<0.001	0.0002	0.10

The most commonly performed procedures were adhesiolysis (53.7%), ovarian cystectomy (30%), ovarian drilling (8.7%), and salpingectomy (7.5%). A comparison of VAS scores in both the intervention and control groups was also done in the most commonly performed procedures. VAS score was measured at all three sites in patients undergoing adhesiolysis in both groups, and the results were compared (Table 3). 

**Table 3:** VAS scores in cystectomy patients at 2, 4, 12, 24 hours, and 1 week for both groups. VAS: Visual analog scale.

Group		VAS 2h	VAS 4h	VAS 12h	VAS 24h	VAS 1 week
Shoulder tip						
I	N	21	21	21	21	21
	Mean [±SD]	5.4 [±0.7]	4.5 [±0.6]	2.5 [±0.9]	2.0 [±0.8]	1.3 [±0.4]
II	N	22	22	22	22	22
	Mean [±SD]	5.6 [±0.7]	4.9 [±0.7]	3.8 [±0.7]	2.3 [±1.0]	1.5 [±0.5]
Results	P-value	0.36	0.05	<0.001	0.20	0.27
At abdomen						
I	N	21	21	21	21	21
	Mean [±SD]	6.7 [±0.5]	5.0 [±0.5]	3.1 [±0.5]	2.0 [±0.8]	1.3 [±0.4]
II	N	22	22	22	22	22
	Mean [±SD]	6.6 [±0.4]	5.3 [±0.6]	3.9 [±0.7]	2.5 [±1.0]	1.5 [±0.5]
Results	P Value	0.42	0.05	<0.001	0.09	0.16
Port sites						
I	N	21	21	21	21	21
	Mean [±SD]	5.8 [±0.6]	5.2 [±0.4]	2.5 [±0.9]	1.5 [±0.5]	1.2 [±0.4]
II	N	22	22	22	22	22
	Mean [±SD]	6.0 [±0.7]	5.4 [±1.0]	3.9 [±0.8]	2.4 [±0.5]	1.5 [±0.5]
Results	P-value	0.37	0.62	<0.001	<0.001	0.15

In all measured areas, Group I (the intervention group) consistently showed a lesser mean VAS score compared to Group II at various time intervals.

The mean value of the VAS score at the shoulder tip at 12 hours was found to be statistically significant, with a p-value of <0.001. The mean value of the VAS score at the abdomen except port sites at 12 hours was found to be statistically significant with a p-value of <0.05. The mean value of the VAS score at port sites at 2 hours after completion of surgery at 12 hours and 24 hours was found to be statistically significant with a p- value of <0.05. VAS score was measured at all three sites in patients undergoing cystectomy in both groups, and the results were compared (Table 4). 

**Table 4:** VAS scores at shoulder tip in cystectomy patients at 2, 4, 12, 24 hours, and 1 week in both groups. VAS: Visual analog scale.

Group		VAS 2h	VAS 4h	VAS 12h	VAS 24h	VAS 1 week
Shoulder tip in cystectomy						
I	N	11	11	11	11	11
	Mean [±SD]	6.5 [±0.5]	5.4 [±0.6]	4.1 [±0.9]	1.9 [±0.7]	1.5 [±0.5]
II	N	13	13	13	13	13
	Mean [±SD]	7.3 [±0.9]	6.2 [±0.8]	4.5 [±1.1]	2.6 [±0.8]	1.3 [±0.4]
Results	P-value	0.01	0.02	0.42	0.04	0.25
At abdomen excluding port sites						
I	N	11	11	11	11	11
	Mean [±SD]	7.2 [±0.6]	5.5 [±0.5]	3.9 [±0.7]	1.9 [±0.7]	1.3 [±0.5]
II	N	13	13	13	13	13
	Mean [±SD]	7.5 [±0.6]	6.1 [±0.6]	4.6 [±0.9]	2.6 [±0.8]	1.3 [±0.4]
Results	P-value	0.33	0.02	0.05	0.04	0.78
Port sites						
I	N	11	11	11	11	11
	Mean [±SD]	6.5 [±0.5]	5.8 [±0.6]	4.1 [±0.9]	2.2 [±0.9]	1.2 [±0.4]
II	N	13	13	13	13	13
	Mean [±SD]	7.3 [±0.7]	6.4 [±0.9]	4.6 [±1.0]	2.3 [±0.7]	1.2 [±0.4]
Results	P-value	0.005	0.06	0.22	0.74	0.82

At 2 hours after the completion of surgery, the mean VAS score for shoulder tip pain in Group I was 6.5 (±0.5) compared to 7.3 (±0.7) in Group II. This difference was statistically significant with a p-value of 0.01 (P<0.05). Similarly, there is a significantly lower VAS score at 4 hours and 24 hours. Group I (intervention group) had a lesser mean VAS score at 2, 4, 12 and 24 hours compared to Group II. The mean value of the VAS score at the shoulder tip at 2 hours after completion of surgery at 4 and 24 hours was significantly less in Group I with p-value <0.05. Group I (intervention group) had a lesser mean VAS score at 2, 4, 12, and 24 hours than Group II. The mean value of the VAS score at the shoulder tip at 2 hours after completion of surgery in Group I was 6.5 (±0.5), whereas in Group II, it was 7.3 (±0.7) with a p-value of 0.005, which was statistically significant. Group I (intervention group) had a lesser mean VAS score at 2, 4, 12, and 24 hours compared to Group II. In Group I, six patients needed rescue analgesia; in Group II, 18 patients required rescue analgesia. Injection of diclofenac sodium 75 mg intramuscular was given as rescue analgesia. The p-value was 0.007, which was statistically significant.

## Discussion

Laparoscopy, being a minimally invasive surgery, offers various advantages over open procedures, but postoperative pain is still considerable. Pain is an essential factor in terms of patient satisfaction, early ambulation, and overall recovery. One of the hypotheses for pain is that CO2, introduced in the peritoneal cavity to create pneumoperitoneum, creates an acid milieu by dissolution of CO2 gas, which causes phrenic nerve damage and peritoneal irritation [3,6]. Various methods have been tried to understand the cause of pain and strategies to reduce it. Use of opioids, NSAIDs, aspiration of gas or using gas drain under the diaphragm, instillation of local anesthetic agent intraperitoneally, and infiltration of local anesthetic on the skin are some of them [4]. As CO2 might have a key role in causing postoperative pain, washing out or neutralizing CO2 may have a role in reducing postoperative pain [6]. It has been postulated that bicarbonate can reduce postoperative pain by neutralizing the acidic peritoneal environment created by CO2 and also prevents phrenic nerve damage and peritoneal irritation. In the present study, we aim to study the effect of sodium bicarbonate in reducing postoperative pain in levels 1 and 2 operative laparoscopic surgeries. The age range in the present study was comparable to other similar studies in the past. In the present study, the mean age ± SD of age in Group I (intervention group) was 28.5 ± 4.96 years, and the corresponding value in Group II (control group) was 30.4 ± 7.04 years. In a study performed by Saadati K et al. [7] in which intraperitoneal irrigation with normal saline and sodium bicarbonate was done in patients undergoing laparoscopic cholecystectomy, the mean age of the intervention group and control group were 44.98 and 42.11 years, respectively. Similarly, another study conducted by Butala BP et al. [9] examined the efficacy of intraperitoneal bupivacaine, with or without morphine, in gynecological laparoscopic surgeries for pain assessment. The mean ages of the intervention and control groups in this study were 35.63 and 34.3 years, respectively. In a study by Roy KK et al. [10] that investigated whether intraperitoneal bupivacaine effectively reduces postoperative pain in individuals with infertility undergoing diagnostic minilaparoscopy, the mean ages of the intervention and control groups were 28.4 and 29.3 years, respectively.

The parity in the present study was comparable to other similar studies. There was no significant difference in parity in both groups, with a p-value of 0.62. In a study conducted by Manjunath AP et al., the efficacy of intraperitoneal lignocaine was seen in women undergoing laparoscopic tubal ligation [11]. The intervention group received 20 ml of 0.5% lignocaine, and the placebo group received 20 ml of isotonic saline intraperitoneally. The maximum number of patients in the intervention and control groups had a parity of 2. Most patients were operated on for infertility, while others had ovarian cysts, chronic ectopic, hydrosalpinx, or polycystic ovarian syndrome. Operative laparoscopy was performed. In research conducted by Butala BP et al. [9], the primary reasons for undergoing gynecological laparoscopic surgery were operative laparoscopy for ovarian cysts and laparoscopic-assisted vaginal hysterectomy. In contrast, a study by Roy KK et al. focused on using diagnostic laparoscopy to address infertility issues. In the present study, when considering both Groups I and II, 53% of patients underwent adhesiolysis, and 30% underwent cystectomy. Patients with a past history of any surgery or caesarean delivery were noted. 11% of the patients had a history of previous surgery. In the current study, the mean operating time for Group I (intervention group) was 30.5 ± 3.9 minutes, and for Group II (control group), it was 31.7 ±5.6 minutes. In a study performed by Rajneesh K et al., where intraperitoneal saline wash was administered to patients undergoing laparoscopic cholecystectomy, the mean operating time was 74.25 ± 22.20 (35-120 minutes) for the intervention group and 66.25 ± 25.89 (25-110 minutes) for the control group [12]. Sugihara M et al. conducted a study wherein 2 mL of 0.5% levobupivacaine was injected at the end of the laparoscopic surgery using a 25G needle for every cm of wound into the muscular fasciae [13]. Surgeries were performed for benign gynecologic diseases. The operation time for the intervention and control groups in this study was 84 ± 50.3 minutes and 75.0 ± 51.0 minutes, respectively. The operating time in the present study was comparable to other studies. This study was performed to compare the effect of intraperitoneal irrigation with sodium bicarbonate and normal saline in operative laparoscopy to reduce postoperative pain in Group I and Group II, of which Group I was composed of patients with intraperitoneal irrigation with normal saline and Group II was composed of patients with intraperitoneal irrigation with sodium bicarbonate after operative laparoscopy. The severity of pain was recorded using a VAS score (0 indicating no pain and 10 indicating unbearable pain) at 2, 4, 12, 24 hours, and 1 week after surgery. Pain was recorded at three sites, which includes shoulder tip pain, abdomen excluding port site, and port site. Patients who required additional analgesics were given injections of diclofenac sodium 75 mg IM for pain relief, and this was recorded in the patient proforma. The mean value of the VAS score at the shoulder tip at 2 hours after completion of surgery in Group I was 5.8 ± 0.8, whereas in Group II, it was 6.3 ± 1.1 with a p-value of 0.02, which was statistically significant. Similarly, the mean values of VAS score at 4 and 12 hours were calculated and compared between the two groups and found to be statistically significant P<0.05. The mean value of the VAS score at 24 hours after surgery in Group I was 2.0 ± 0.7, whereas in Group II, it was 2.4 ± 0.9 with a p-value of 0.06, which was not statistically significant. Similarly, the mean values of VAS score at 1 week were calculated and compared between the two groups and found to be statistically insignificant (p<0.05). Group I (intervention group) had lesser mean VAS scores at 2, 4, 12, 24 hours, and 1 week compared to Group II. The mean value of the VAS score at the abdomen excluding port site at 2 hours after completion of surgery in Group I was 6.9 ± 0.5. In contrast, in Group II, it was 6.9 ± 0.6 with a p-value of 0.85, which was statistically insignificant. The mean value of the VAS score at 4 hours in Group I was 5.2 ± 0.6, whereas in Group II, it was 5.6 ± 0.7 with a p-value of 0.003, which was statistically significant. Similarly, the mean VAS score at 12 hours and 24 hours after surgery was less in the intervention group, and the difference was statistically significant. The mean value of the VAS score at the port site two hours after completion of surgery in Group I was 6.1 ± 0.6, whereas in Group II, it was 6.5 ± 0.9 with a p-value of 0.01, which was statistically significant. Similarly, the mean values of VAS score at 12 hours and 24 hours after surgery were less in the intervention group, and the difference was statistically significant. The mean VAS score in two procedures, i.e., adhesiolysis and cystectomy at all three sites, was comparable, and no difference was seen in the VAS score between the two. No adverse effects were seen with the use of sodium bicarbonate. Therefore, intraperitoneal irrigation with sodium bicarbonate was found to be effective in reducing postoperative pain. Similar studies were done to assess VAS scores with the use of different interventions by Saadati K et al. [7], Rajneesh K et al. [12], Roy KK et al. [10], and Butala BP et al. [9], as discussed below.

In a study conducted by Saadati K et al., the efficacy of sodium bicarbonate in postoperative pain relief following laparoscopic cholecystectomy was assessed and compared to both a normal saline group and a no-washing group [11]. The researchers observed a significant reduction in pain at the shoulder tip in the sodium bicarbonate group compared to the non-washing Group at 6, 18, and 24 hours postoperatively, with p-values of 0.04, 0.02, and 0.009, respectively. However, no significant differences in pain were noted at other sites, including the right shoulder tip, back, and port site. Another study was done by Rajneesh K et al. to study the effect of intraperitoneal saline wash in laparoscopic cholecystectomy [12]. Two groups were made; Group 1 received intraperitoneal saline, and Group 2 did not receive it. Shoulder tip pain was assessed postoperatively in saline irrigation versus non-irrigation Group, and it was found that pain was less in the irrigation group, although the difference in pain score was not significant. At 1 hour, 6 hours, and 6-12 hours following surgery, Group 1 had a considerably reduced need for further analgesics (P=0.005, P=0.000, and P=0.005, respectively). The authors concluded that intraperitoneal saline wash was useful in reducing shoulder tip pain following laparoscopic cholecystectomy. In the double-blinded randomized study done by Butala BP et al. in women undergoing laparoscopic gynecological surgeries [9], the efficacy of bupivacaine instilled intraperitoneally was compared with morphine use. Postoperative pain was assessed using the VAS score. A total of 90 women were randomized into three groups, each with 30 patients. In Group BM, both bupivacaine and morphine were instilled intraperitoneally. In BO group, only bupivacaine, and in the third group, saline was injected before the removal of trocar at the end of surgery. In the bupivacaine and morphine group, a significant reduction in immediate postoperative pain was seen (VAS: 23.33 ± 6.04 versus 45.5 ± 8.57). Also, there was reduced pain four hours after surgery in the BM group (VAS 24 ± 12.13 versus 41.17 ±7.27 in the BO group). The authors concluded that along with local anesthetic, the addition of morphine significantly reduced postoperative pain and the need for rescue analgesia. There was no significant increase in side effects.

Roy KK et al. conducted a prospective randomized study to evaluate the efficacy of intraperitoneal bupivacaine in reducing postoperative pain after diagnostic mini-laparoscopy in patients with infertility [10]. The treatment group (Group A) received 10 mL of intraperitoneal 0.25% bupivacaine (100 mg) at the end of the operation, whereas the control group (Group B) received 10 mL of intraperitoneal saline. Postoperative pain was quantified using a VAS ranging from 1 to 10, with assessments made at 2, 4, 6, and 8 hours post-surgery. The intervention group's pain scores were significantly lower at 2, 4, 6, and 8 hours following the procedure (P<0.05). The authors drew the conclusion that intraperitoneal bupivacaine significantly lessens pain for up to 8 hours after surgery and lessens the need for additional analgesics. The mean VAS score at the first 24-hour post-surgery calculated in our study was statistically significant at the shoulder tip, abdomen excluding the port site, and port site. A similar study performed by Saadati K et al. [7] showed that postoperative pain at the left shoulder tip was reduced with sodium bicarbonate irrigation, and the difference was statistically significant. Hence, our study shows that the intervention group had significant benefits in terms of postoperative pain. The present randomized control trial demonstrated that intraperitoneal irrigation with sodium bicarbonate was feasible and safe for postoperative pain relief. Using this method at the end of procedures in women undergoing operative laparoscopy led to a significant reduction in the VAS score, without any impairment in surgical outcomes. The results were consistent across different procedures, specifically laparoscopic cystectomy and adhesiolysis. The study had a few limitations. It was a single-center randomized controlled trial with participants from the same ethnic background. The sample size was limited due to the prevailing COVID-19 pandemic at the time of trial recruitment, as most laparoscopic procedures were abandoned during that period.

## Conclusions

In the present study, the mean value of the VAS score at the shoulder tip was significantly reduced in the intervention group at 2, 4, and 12 hours after surgery. The mean value of the VAS score at the abdomen except port sites was found to be significantly reduced in the intervention group at 4, 12, and 24 hours after surgery. The mean value of the VAS score at port sites was found to be significantly reduced in the intervention group at 2, 12, and 24 hours after surgery. With an increase in the time period from surgery, the mean value of the VAS score decreased in both groups. However, the mean values of the VAS score were lower in the intervention group. When the mean value of the VAS score was compared across different procedures, similar outcomes were found. Therefore, intraperitoneal irrigation with sodium bicarbonate proves beneficial in reducing postoperative pain during operative laparoscopy. Nevertheless, multicenter trials with a larger number of participants would be useful to confirm these findings.
